# Autologous cord blood mononuclear cell infusion for the prevention of bronchopulmonary dysplasia in very preterm monozygotic twins: A study protocol for a randomized, placebo-controlled, double-blinded multicenter trial

**DOI:** 10.3389/fped.2022.884366

**Published:** 2022-12-09

**Authors:** Ren Zhuxiao, Huang Ruoyu, Yang Liling, Ren Xuejun, Yang Chunhui, Ruan Wanfen, Chen Zhifeng, Dai Yiheng, Zhang Qi, Wei Wei, Liu Zhipeng, Pei Jingjun, Yin Qigai, Yang Jie

**Affiliations:** ^1^ Department of Neonatology, Guangdong Women and Children Hospital, Guangzhou Medical University, Guangzhou, China; ^2^ Department of Neonatology, The First Affiliated Hospital of Kangda College of Nanjing Medical University, Nanjing, China; ^3^ Department of Neonatology, Dongguan Maternal & Child Health Hospital, Dongguan, China; ^4^ Department of Neonatology, Zhongshan Boai Hospital, Zhongshan, China; ^5^ Department of Neonatology, Shunde Hospital, Southern Medical University, Foshan, China; ^6^ Department of Neonatology, Dongguan Hospital, Southern Medical University, Dongguan, China; ^7^ Department of Neonatology, Affiliated Maternal & Child Health Hospital of Foshan, South Medical University, Foshan, China; ^8^ Department of Clinic Genetic Center, Guangdong Women and Children Hospital, Guangzhou Medical University, Guangzhou, China; ^9^ Guangdong Cord Blood Bank/Guangzhou Municipality Tianhe Nuoya Bio-Engineering Co. Ltd, Guangzhou, China; ^10^ Department of Neonatology, Nanfang Hospital, Southern Medical University, Guangzhou, China

**Keywords:** cord blood mononuclear cells, bronchopulmonary dysplasia, very preterm monozygotic twins, prevention, autologous, study protocol

## Abstract

**Background:**

Preterm-associated complications remain the main cause of neonatal death. Survivors face the challenges of short- and long-term complications. Among all complications, bronchopulmonary dysplasia (BPD) remains the first important cause of neonatal mortality and morbidity. Current treatment does not address this main preterm complication. Cord blood is regarded as a convenient source of stem cells. The paracrine bioactive factors of stem cells contribute to tissue repair and immune modulation. Our clinical studies and those of others have shown that cord blood cell infusion is both safe and possibly effective in the prevention and treatment of BPD. The therapeutic use of cord blood has emerged as a promising therapy. However, the genetic heterogeneity between control and intervention groups may reduce the comparability especially among small sample trials. The purpose of this study protocol is to investigate the effects of autologous cord blood mononuclear cell (ACBMNC) infusion on the prevention of BPD in very preterm monozygotic twins of less than 32 gestation weeks.

**Methods:**

In this prospective, randomized, placebo-controlled, double-blinded multicenter clinical trial, 60 pairs of monozygotic twin preterm neonates of less than 32 weeks admitted to the Neonatal Intensive Care Unit are randomly assigned to receive intravenous ACBMNC infusion (targeted at 5 × 10^7^ cells/kg) or placebo (normal saline) within 24 h after birth in a 1:1 ratio. The primary outcome will be survival without BPD at 36 weeks of postmenstrual age. The secondary outcomes will include the mortality rate, BPD severity, other common preterm complication rates, respiratory support duration, length and cost of hospitalization, and long-term respiratory and neurodevelopmental outcomes during a 2-year follow-up. Furthermore, we will perform single-cell RNA sequencing for cord blood cells and blood cells 3–10 days after intervention and detect whether reactive oxygen species and inflammatory cytokines are present.

**Conclusion:**

This will be the first randomized, placebo-controlled, double-blinded trial to evaluate the efficacy of ACBMNC infusion to prevent BPD in monozygotic twin premature infants and investigate the underlying protective mechanisms. The results of this trial will provide valuable clinical evidence for translational application of cord blood cell therapy in very preterm infants.

**Trial registration:** ClinicalTrials.gov, NCT05087498, registered 10/09/2021, https://register.clinicaltrials.gov/prs/app/action/SelectProtocol?sid=S000BAD7&selectaction=Edit&uid=U0002PLA&ts=2&cx=qvyylv.

## Introduction

Preterm births and their complications are the leading cause of death for children under 5 years old (1). Preterm-associated lifetime disability, including intellectual and physical disability, and chronic lung disease, contributes to a huge social burden (2–4). Intensive care therapy in the Neonatal Intensive Care Unit (NICU) has seen great advances in recent years; however, the long-term morbidity rate has not shown any improvement. Inversely, given the higher survival rates of very preterm neonates, the incidence of bronchopulmonary dysplasia (BPD) increases (2, 5, 6). Fragile preterm neonates experience persistent tissue injury and insufficient repair because of their immature organs and various invasive therapies that they are subjected to after birth (6, 7). The immature lung is the main organ that is commonly affected. Alveolar simplification and dysregulated vascularization are the main characteristics of BPD (3, 7, 8). Current treatments do not address the underlying pathological process of this main preterm complication (3, 5, 9).

Stem cell therapy has shown promising potential in supporting tissue development and cell repair and is rapidly emerging as a novel therapeutic tool for several neonatal diseases (10–12). Human umbilical cord blood cells (HUCBCs) are abundant in stem cells (13, 14). These primitive cells can produce anti-inflammatory, anti-apoptotic, and immunoregulatory bioactive factors through paracrine effects and stimulate endogenous cell repair (15, 16). Preclinical studies have demonstrated the effect of cord blood stem cell infusion on the prevention and treatment of BPD (15, 17). Evidence from our clinical trials and the trials of several others has proved the safety, feasibility, and potential effect of umbilical cord blood (UCB)-derived stem cell administration in preterm neonates as well as long-term safety (18–21). However, the current trials often suffer from a small sample size, and the final statistical analysis results of the main outcomes show no significant difference between the cord blood cell intervention group and the control group (18–21).

Both genetic predisposition and intervention difference between individuals affect the clinical outcomes of patients (6, 22). To increase the statistical potency of the therapeutic effect of cord blood stem cells especially among small sample trials, enrolling very preterm monozygotic twins of less than 32 gestation weeks (GW) could improve the comparability between intervention and control groups as the hereditary material is the same within monozygotic twins.

With these concepts in mind, here, the authors will enroll very preterm monozygotic twins and assign the random number. Therefore, one of the twins will be assigned to the control group and the other to the intervention group. Furthermore, we will perform single-cell RNA sequencing for cord blood cells and blood cells 3–10 days after intervention and detect whether reactive oxygen species and inflammatory cytokines are present in serum from abandoned blood samples. As the genetic background is the same within monozygotic twins, the results of single-cell RNA sequencing are expected to have better comparability, following which the mechanisms elucidated will reveal the effect of autologous cord blood mononuclear cell (ACBMNC) infusion to the maximum (23). This will be the first randomized, placebo-controlled, and double-blinded trial to evaluate the efficacy of ACBMNC infusion as a prophylactic measure for BPD in monozygotic twin premature infants who have the same genetic background and investigate the underlying protective mechanisms *via* single-cell RNA sequencing.

## Methods

### Study design and settings

**Figure 1:**
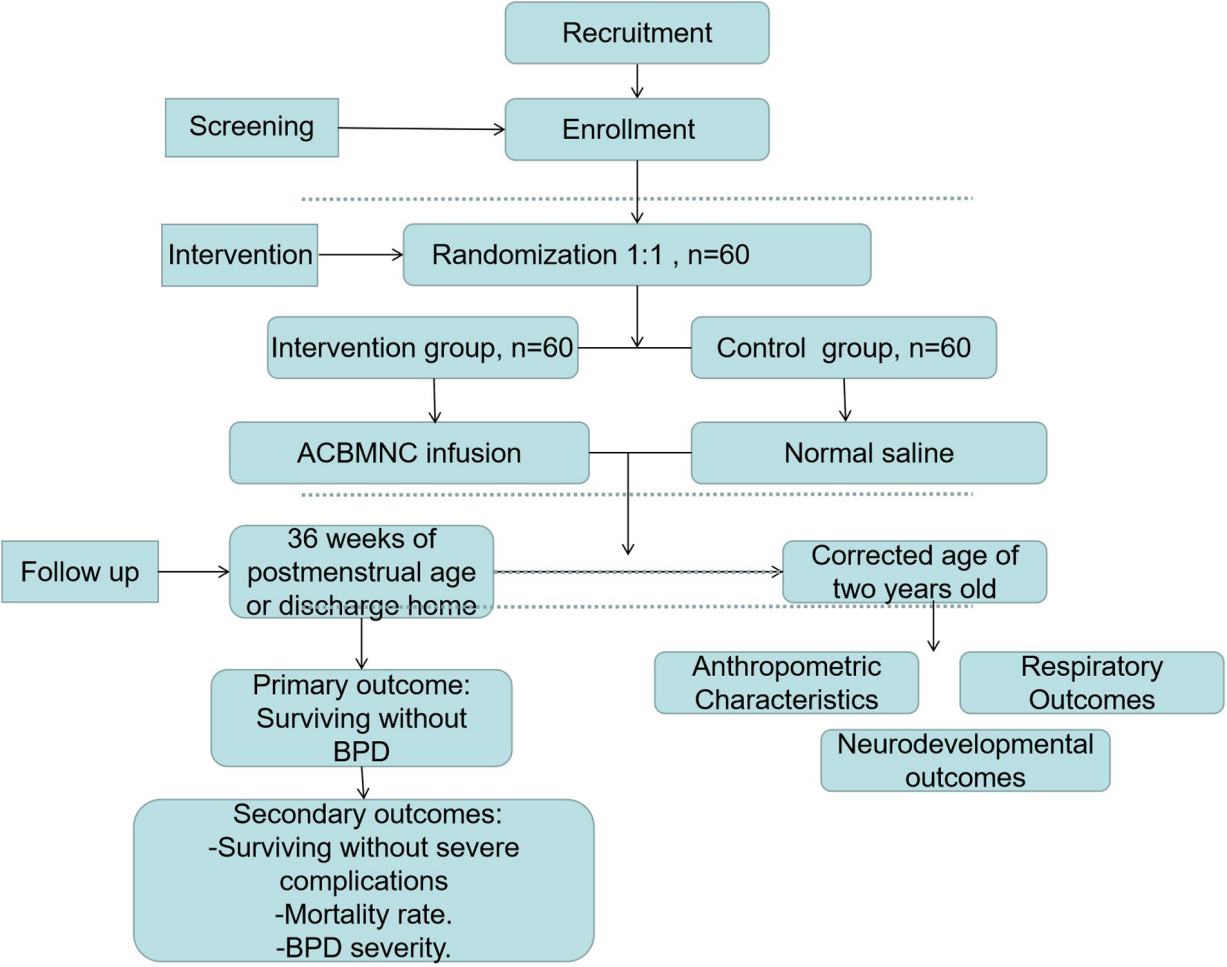
Consort flow diagram.

This study protocol describes a randomized, placebo-controlled, double-blinded, and multicenter trial to be conducted at seven medical centers (Guangdong Women and Children Hospital, Dongguan Maternal and Child Health Hospital, Zhongshan Boai Hospital, Shunde Hospital-Southern Medical University, Dongguan Hospital-Southern Medical University, Nanfang Hospital-Southern Medical University, and Affiliated Maternal and Child Hospital of Foshan-Southern Medical University) in tertiary hospitals. The participating NICUs are selected by an expert committee based on the distance to the Guangdong Cord Blood Bank (GDCBB) and the level of intensive care that these NICUs could provide. The Guangdong Cord Blood Bank is a public provincial blood bank affiliated with the Guangdong Women and Children Hospital and collects cord blood routinely at these enrolled hospitals. To ensure that the cord blood is processed and infused to infants within 24 h after birth, the distance between these centers should be short. Furthermore, to reduce the differences in therapeutic treatment between the selected centers, a uniform guideline regarding the treatment of patients has been devised in all clinical centers. These centers hold quality control meetings every 3 months to ensure research consistency and report and analyze the possible side effects caused by MNC infusion. In this study, a total of 60 pairs of monozygotic twin premature infants fulfilling the eligibility criteria will be enrolled. For each pair, one random number will be assigned to the first baby of the twins; if the last digit of this random number is odd, the first baby will be assigned to the intervention group, and the second baby will be automatically assigned to the control group; in contrast, if the last digit of this random number is even, the first baby will be assigned to the control group, and the second will be automatically assigned to the intervention group. The protocol for this study has been developed based on the Standard Protocol Items: Recommendations for Interventional Trials (SPIRIT) checklist (Supplementary material S1, S2). We have followed the Consolidated Standards of Reporting Trials (CONSORT) guidelines and the study design is illustrated in Figure 1.

## Trial objectives

### Primary objective

The primary objective of this trial is to evaluate the efficacy of ACBMNC infusion for the prevention of BPD in very preterm monozygotic twins of less than 32 GW.

### Secondary objectives

The secondary objectives of this trial are as follows: (1) to compare the infant mortality rate at 36 weeks of postmenstrual age; (2) to compare the rates of other common preterm complications such as intraventricular hemorrhage (IVH), necrotizing enterocolitis (NEC), retinopathy of prematurity (ROP), respiratory distress syndrome (RDS), ventilation-associated pneumonia (VAP), hypoxic ischemic encephalopathy (HIE), late-onset sepsis (LOS), periventricular leukomalacia (PVL), and anemia; (3) to compare the duration of mechanical ventilation and oxygen therapy in the two groups; (4) to determine the re-intubation rate and time of return to birth weight (BW); (5) to compare the duration of antibiotic usage; (6) to determine the long-term outcomes during a 2-year follow-up, including anthropometric characteristics, respiratory outcomes, and neurodevelopmental outcomes *via* a standardized neurological examination; (7) to compare the results of single-cell RNA sequencing for cord blood cells and blood cells 3–10 days after intervention and detect whether reactive oxygen species and inflammatory cytokines are present in serum.

## Participants

### Inclusion criteria

Infants fulfilling all the following inclusion criteria will be enrolled in this trial: (1) infants born at study hospitals; (2) monozygotic twins (this will be confirmed by ultrasonographic diagnosis before birth and confirmed again with obstetricians during and after delivery, as monochorionic diamniotic twins and monochorionic monoamniotic twins are both monozygotic twins; therefore, both will be eligible) (24); (3) gestational age (GA) < 32 weeks (GA was calculated based on the date of the last menstrual period of the mother and an ultrasonographic screening performed during the first trimester of pregnancy); (4) those enrolled within the first 24 postnatal hours; (5) those free of severe perinatal asphyxia (defined as an Apgar score of 0–3 for more than 5 min, a cord blood gas pH < 7.00, or both); (6) those free of severe congenital anomalies or genetic syndromes; (7) those free of mother infections such as chorioamnionitis, TORCH syndrome (chorioamnionitis, TORCH syndrome, were diagnosed as previously described (24, 25); (8) written informed consent is obtained from the parents or guardians of the infants; (9) either of the twins has available UCB, and the cell number should be at least 5 × 10^7^ cells per kilogram.

### Exclusion criteria

Infants will be excluded from the study if (1) they exhibit severe congenital abnormalities (detected *via* prenatal ultrasound); (2) their condition is critical and are expected to die within the first 24 h; (3) they are diagnosed with twin-to-twin transfusion syndrome confirmed by prenatal ultrasonography (24).

### Ethical approval

This study is approved by the ethics committee of the Guangdong Women and Children Hospital. The ethics number is 202101265.

### Sample size

Our previous study showed that ACBMNC infusion was effective in reducing respiratory support duration in preterm infants (18). A randomized study in Korea by Won Soon Park showed that severe BPD incidence significantly reduced from 53% to 19% with mesenchymal stem cell intervention in the 23–24 GW group (*n* = 33) (20). The rate of BPD among very preterm infants at our NICU was 30% (pA), which is similar to the incidence rate reported previously (26). The sample size of this study was determined for assessing the efficacy of ACBMNCs compared with placebo with respect to the primary outcome in order to decrease the incidence of BPD or death with a two-sided α level of 0.05% and 80% power. We expect the intended (or at least acceptable) effect of ACBMNC infusion to be at a rate of 20% decrease in the frequency of BPD. To achieve this rate, at least 60 patients per group will be required based on the following formula:

n=(pA(1−pA)/κ+pB(1−pB))((z1−α/2+z1−β)/(pA−pB))2)

κ = nA/nB is the matching ratio; α is type I error; β is type II error, meaning 1 − β is power.

Around 20 pairs of monozygotic twin births of less than 32 gestational weeks occur annually in Guangdong Women and Children Hospital and Dongguan Maternal and Child Health Hospital. Around 6 pairs of monozygotic twin births of less than 32 gestational weeks take place annually in Zhongshan Boai Hospital, Shunde Hospital, Southern Medical University, Dongguan Hospital, Southern Medical University, Nanfang Hospital, Southern Medical University, and Affiliated Maternal and Child Hospital of Foshan, Southern Medical University. Therefore, we assume that 60 pairs of monozygotic twins will be enrolled annually and the trial could be completed within 1 year.

### Randomization

The randomization sequence will be generated electronically using SPSS (version 21). Following enrollment, the subjects will be assigned to treatment levels or groups after the verification of their eligibility and consent status. A random number will be assigned by computer for each pair of enrolled twins. For each pair, one random number will be assigned to the first baby of the twins; if the last number of this random number is odd, the first baby will be assigned to the intervention group, and the second baby of the twins will be automatically assigned to the control group; in contrast, if the last number of this random number is even, the first baby of the twins will be assigned to the control group, and the second will be automatically assigned to the intervention group. Those enrolled in the ACBMNC group will receive an infusion of ACBMNC within 24 h after birth. Those in the placebo group will receive an infusion of a placebo solution consisting of normal saline with similar volume based on the cell number results in their cord blood after processing. The ell dose for all patients will be 5 × 10^7^ cells per kilogram (18, 27).

### Blinding

**Figure 2:**
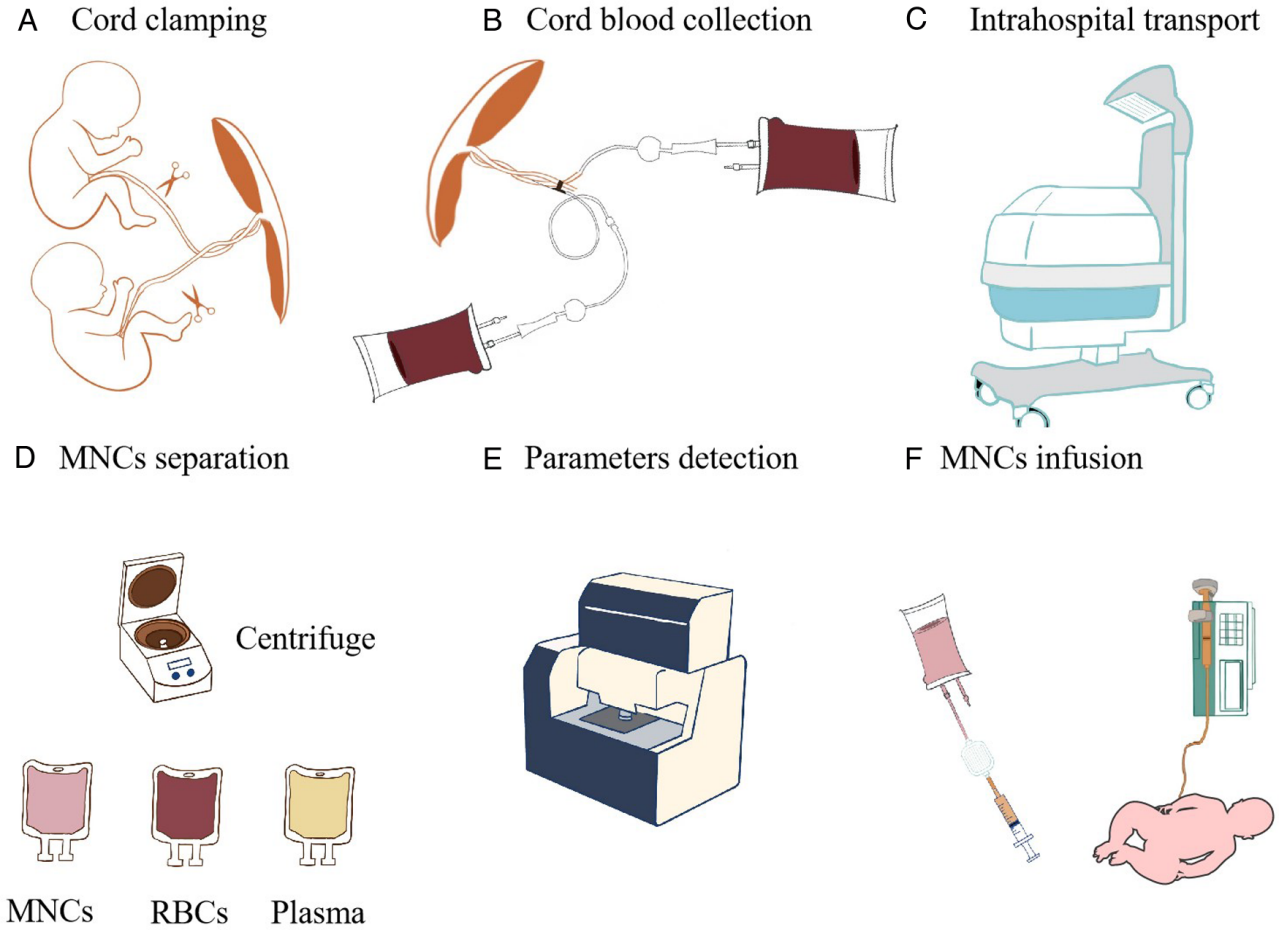
Cord blood processing flow diagram. (**A**) Cord clamping. (**B**) Cord blood collection. (**C**) Intrahospital transport. (**D**) MNC separation after centrifugation. (**E**) Parameter detection. (**F**) MNCs are infused by using a light-blocking infusion tube and placing an IV line vertically.

The study data will be reviewed by the hospital ethics committee during the trial. Nobody will be made aware of the treatment-group assignments of the infants. After the enrollment is confirmed, the research assistants of the respective project leaders will confirm the random number. There will be at least one research nurse and physician in each center. Research nurses and physicians who are aware of the allocation of treatment will not be included in the babies’ care team and they will not participate in making care decisions for the babies in the trial. They will be asked to prepare the processed cord blood MNC or normal saline. Infusion bags will be covered with shading bags and MNCs will be infused by using a light-blocking infusion tube after preparation, so that the nurses who carry out the infusion and all physicians who treat the baby are not aware of the treatment assignment (Figures 2B,C). The infusion is then done by the supervisor nurse of the patients. The staff who collect and analyze the patients’ data and who follow up the patients are also blinded with the assignment. What is more, the infants’ parents are also not aware of the assignment. Therefore, this study is a double-blinded one.

## Intervention

### Enrollment and umbilical cord blood collection and transportation

Written informed consent is obtained from the parents or guardians of all infants before delivery. For each monozygotic twin, neonatologists will be summoned at least 1 h before delivery, and their brief is to study patient data and confer with the obstetrician to verify whether the patients fulfill the enrollment criteria. An intrahospital transport team will be organized, which includes three neonatologists (one of them will be a senior physician). A transport incubator and ventilator with an air oxygen mixer and heater humidifier that can provide intermittent positive-pressure ventilation will be equipped for intrahospital transport from the delivery suite to the NICU in the same hospital (Figure 2C). Two blood-collection bags (WEGO, China) containing 28 ml of citrate-phosphate-dextrose anticoagulant and several blood-collection tubes are prepared.

After the neonates are delivered, delayed cord clamping is done for 30–60 s if the babies do not show severe asphyxia and do not need immediate resuscitation. After the twins are born and before the placenta is delivered, both umbilical veins are sterilized and then punctured with a 17-gauge needle. A part of the cord blood will be collected to the blood-collection tubes for clinical blood tests and single-cell RNA sequencing within 24 h after collection, and then cord blood is collected using a blood-collection bag (WEGO, China) by trained medical staff (Figures 2A,B).

Enrollment will be evaluated again by the neonatologists according to the inclusion and exclusion criteria. The blood-collection bag is closed and sealed after the collection is completed. The cord blood bag is labeled with the full name of the neonate, the hospital name, and the birth time and then placed in a cord blood storage container at 4°C storage and sent to the GDCBB within 2 hours. The GDCBB is a provincial, public cord blood bank with the clinical certification of the American Association of Blood Bank (AABB) and authentication of the Chinese Ministry of Health. The GDCBB has been equipped with a thermostat that is specifically used for cord blood transportation; it is able to maintain a temperature of 4°C during transportation. The transportation time duration (both from and back to the hospital) is within 2 hours depending on the traffic condition.

### Cord blood processing

Procedures for cord blood processing will be performed in accordance with cord blood bank guidelines as described previously (28).

Briefly, after arriving at the GDCBB, the blood bags are transported to a 10000-grade aseptic operational workshop *via* a transmission window. Two milliliter of cord blood is taken to detect viruses [human immunodeficiency virus, hepatitis B virus, hepatitis C virus, cytomegalovirus by polymerase chain reaction (PCR)] and bacterial infections (including *Treponema pallidum-*antibody detection or blood smear). The results are obtained shortly before the transfusion is started. The blood bags are then centrifuged at 4°C with a gravity of 50 g for 8 min in a low-temperature centrifuge (RT, Beckman, United States). Then, red blood cells are removed. After that, the blood bags are turned straight and centrifuged for 10 min in the same condition as before. Then, MNCs are obtained after the plasma is separated by using the plasma separation clip (Figure 2D). Finally, a 1 ml MNC sample is taken to determine the following parameters: MNC count (Sysmex XE-5000 automated flow cytometer, Japan), CD34 count (BD calibur flow cytometry, BD Bioscience, United States), colony-forming unit-granulocyte and macrophage (CFU-GM) count under an inverted microscope after being cultured in methyl cellulose medium (SIGMA-ALDRICH, United States), sterility detection by an automatic bacteria detector (Thermo Fisher 6240, MA, United States), and cell viability by using the 7-aminoactinomycin D detection kit *via* flow cytometry analysis (Figure 2E). After processing, if the cell number reaches at least 5 × 10^7^ cells per kilogram, the cells will be titrated according to the cell concentration in 5% albumin to ensure that the final infusion volume ranges from 5 to 20 ml/kg and stored in the transparent separation bag in a 4°C refrigerator and then transported in the thermostat used for cord blood transportation. The cord blood cells will be sent back to the hospitals where they are collected. The patients are finally enrolled in the allocation. Even if only one of the twins has enough cord blood for the purposes of study participation, both will be enrolled as part of the randomization process.

### MNC infusion

All infusions will be administered in the NICU. Infusion and subject identities will be double-checked by research nursing and physician staff. Infusions will be monitored by research and clinical staff. ACBMNCs or placebo are administered intravenously *via* a central catheter or a peripheral catheter if a central catheter is not available. ACBMNC infusion should be performed within 4 h of patients arriving in hospital. The maximum volume will be less than 20 ml/kg. An infusion pump is used to control the infusion rate at less than 10 ml/kg/h. A blood filter will be used to filter the cell settings or blood clots before infusion. Prior to infusion, the intravascular (IV) line is prestored with 2 ml MNCs. We will infuse the MNCs by positioning the syringe and IV line vertically (29). During infusion, the nurses will shake the cells gently every half an hour. After infusion, 2-ml normal saline will be used to flush the intravascular catheter (Figure 2F).

### Safety monitoring and co-intervention

Assessment of safety is conducted before, during, and 12 and 24 h after infusion. The heart rate, systolic, diastolic, and mean arterial blood pressure, and arterial blood oxygen saturation level are monitored continually and documented; these are also monitored during the period of hospitalization and return visits. The levels of arterial blood gas and blood glucose in peripheral blood are monitored and kept stable during the whole treatment period. Infusion reactions (fever, hemolysis, hyperbilirubinemia, and urine color changes) and signs of circulatory overload (tachycardia, dyspnea, edema, and hepatomegaly) are observed and checked during infusion and 48 h after infusion.

The trial will be strictly monitored by the members of a safety monitoring board comprising an ethics committee member, project principal, good clinical practice member, and statisticians, and they will be notified of any severe adverse events (including death, LOS, NEC, severe IVH, and cystic periventricular leukomalacia). Other adverse or unexpected events will be reviewed immediately after they are discovered. After each interim analysis (every 3 months), the data safety monitoring board will make a decision on whether to stop or continue the trial based on safety monitoring and sequential analysis of the primary outcome.

All patients in the study will be given intensive care therapy in accordance with uniform guidelines, which include positive-pressure mechanical ventilation, oxygen therapy, and exogenous pulmonary surfactant (PS) (Curosurf, Chiesi, Parma, Italy) replacement. Oxygen saturation targets (90%–95%), ventilatory support, postnatal steroids, and transfusion guidelines are uniformly given and applied to all enrolled patients. Inhaled nitric oxide is given if a patient with severe hypoxemic respiratory failure presents with severe persistent pulmonary hypertension of newborn (PPHN) confirmed by cardiac ultrasound with an estimated peak systolic pulmonary artery pressure that is higher than 35 mm Hg or more than two-thirds of the systemic systolic pressure. Ibuprofen is given to neonates who have significant patent ductus arteriosus (PDA) (28). Postnatal systemic dexamethasone is reserved only for infants who have severe underlying lung disease and intractable respiratory failure. In such infants, a short course of dexamethasone (three to five doses of 0.25 mg/kg every 12 h) is given at the discretion of the attending physician. Chest radiography is performed after the umbilical venous catheter is placed. Arterial blood gas is monitored at least every 24 h until weaning from ventilation. All clinical diagnoses are defined according to a standard reference (29).

### Clinical data collection

The following data will be collected and entered into an online collaborative database:

(1) Basic clinical characteristics: hospital name, patient name, the recorder name, birth date and time, sex, GA, birth weight, height, head circumference, delivery mode, delayed cord clamping time, and Apgar score at 1, 5, and 10 min. Maternal information includes maternal age, race, pregnancy complications, and prenatal glucocorticoid use.
(2) Characteristics of cord blood collection, processing, and infusion: the time between collection (birth) and initiation of infusion, cell number before and after processing, cell concentration before and after processing, MNC infusion starting time, MNC infusion duration, infusion volume, infusion cell number per kg, CFU-GM, CD34 + cell count, and cell viability post processing.
(3) Co-intervention: respiratory support mode during the first 28 days, 36 GW, and discharge home, respiratory support duration, dose of pulmonary surfactant replacement, postnatal steroid use, surgical closure of PDA, use of blood products, any other surgery, inhaled nitric oxide, and antibiotic administration.
(4) Laboratory investigation and image results: peripheral blood glucose before and after infusion, arterial blood gas analysis (PH, PO_2_, PCO_2_) in peripheral blood before and after infusion, serum electrolytes (sodium, potassium, chloride, and calcium), lactic acid, blood urea nitrogen and peripheral blood routine examination (white blood cells, hematocrit, and platelet) during the first 4 weeks of the study, electroencephalogram result, and lung ultrasound during hospitalization.
(5) Single-cell RNA sequencing, reactive oxygen species, and inflammatory cytokines (including IL-10, IL-6, IL-1β, and TNF-α) for cord blood and peripheral blood 3–10 days after infusion (samples will be collected from abandoned blood from routine blood tests only if tests are clinically necessary). Single-cell RNA sequencing, reactive oxygen species, and inflammatory cytokine results between cord blood cells and peripheral blood 3–10 days after intervention in each patient and single-cell RNA sequencing, reactive oxygen species, and inflammatory cytokine results of peripheral blood 3–10 days after intervention between placebo and MNC infusion groups is compared to analyze the therapy-induced molecular signal and immune alteration, therefore exploring the underlying mechanisms of the protective effect of ACBMNC on BPD.

### Outcome measures

Primary outcome:

– Surviving without BPD.

Secondary outcomes:

– Surviving without severe preterm-associated complications (severe BPD, severe ROP, and severe IVH).
– Mortality rate.
– BPD severity.

Other outcomes:

– Incidence of other preterm complications including IVH, PVL, NEC, ROP, RDS, VAP, LOS, and anemia.
– Duration of mechanical ventilation and oxygen therapy.
– Frequency of re-intubation.
– The time (days) to return to BW.
– Body weight during the first 4 weeks (measured on each week).
– Duration of hospitalization.
– Hospitalization expenses.

We will use the following clinical definitions in this study: (all clinical diagnoses will be defined according to a standard reference) (30).

1. GA will be determined on the basis of a combination of the last menstrual period and early ultrasound findings.
2. The diagnosis of common preterm complications will include the following:
  BPD, defined as treatment with oxygen >21% for at least 28 days, and its severity will be assessed at 36 weeks of postmenstrual age or discharge home, whichever comes first. Mild BPD is defined as breathing room air at assessment. Moderate BPD is described as the need for <30% supplemental oxygen, and severe BPD is described as needing ≥30% supplemental oxygen or positive airway pressure. For these patients, BPD status will be analyzed by a committee of three independent experts blinded to the study group.
  RDS will be defined if the infants show evidence of respiratory symptoms such as grunting and chest retraction, typical chest radiography findings, and/or treatment with surfactant, and the need for assisted ventilation.
  NEC will be defined using Bell's classification. Infants with stage II or above will be diagnosed with NEC.
  LOS will be defined if the infants report positive bacterial culture results after the first 72 h of birth.
  ROP will be defined according to the International Classification for Retinopathy of Prematurity. Severe ROP is defined as ROP needing surgical treatment.
  Anemia is defined as hemoglobin of no more than 140 g/L.
  IVH and PVL will be defined by serial head ultrasound, performed according to the description by Volpe. The first head ultrasound will be performed within 3 days after birth and follow-up head ultrasound examinations will be performed every week until the day of discharge. Severe IVH is defined as ≧stage 3 IVH.
  VAP is defined as a pneumonia occurring after the patient has been intubated and has received mechanical ventilation for more than 48 h.

### Follow-up

All infants will be followed up three times (corrected GA of 40 weeks and corrected age of 12 and 24 months), and the following parameters will be recorded.

*Follow-up content at corrected GA of 40 weeks:*

– Main caregiver and the education level.
– Smoking in main caregivers.
– Feeding mode (breastfeeding, formula, and mixed feeding).
– Anthropometric characteristics: weight, length, and head circumference.
– Respiratory outcomes: the occurrence of wheezing, supplemental oxygen requirement, and rehospitalization because of pneumonia.
– Neurodevelopmental outcome: neonatal behavioral neurological assessment score, the occurrence of seizure, encephalomalacia, intracranial hemorrhage, and auditory and visual impairment.
– Results of blood routine examination.

*Follow-up content on corrected age of 12 and 24 months:*

– Anthropometric characteristics: weight, length, head circumference, and anterior fontanel size.
– Respiratory outcomes: the occurrence of wheezing, asthma, nocturnal cough, supplemental oxygen requirement, and rehospitalization because of pneumonia.
– Degree of neurodevelopmental impairment *via* standardized neurological examination (using the Bayley scales) and other major neurodevelopmental outcomes such as cerebral palsy, seizures, auditory impairment, and visual impairment.
– Results of blood routine examination.

### Statistical analysis

Mean and standard deviation are reported for continuous variables, and the number and percentage are reported for categorical variables. Group comparisons of categorical variables for outcomes and co-interventions including death, BPD, and BPD severity are performed using nonparametric tests, the Fisher's exact test, or chi-square test, as appropriate. The relative risk (RR) is calculated as the ratio of the cumulative incidence rates of an event occurring in the intervention infusion group to the control group, and the number needed for treatment as the inverse of these two cumulative incidence rates. The results are shown as RR with 95% confidence interval (CI). For continuous variables, the mean values at each time point in each group of the variables are derived from a generalized linear model and the differences in the values over time are compared using an unpaired Student's t-test between the two groups. Kaplan–Meier cumulative incidence plots are generated to show time-to-event end points, namely, extubation and weaning to room air. All statistical tests are two-tailed, and *p*-values < 0.05 are deemed statistically significant. The Breslow–Day test and Cochran–Mantel–Haenszel (CMH) test will be used to adjust the data derived from different participating centers. The results will be reported as odds ratio (OR) with 95% CI. All statistical tests will be two-tailed and a *p*-value < 0.05 will be considered statistically significant. All statistical analysis will be performed using SPSS 21.0 (IBM).

## Discussion

With the rapid advances in technology in the NICU, the survival rate of preterm infants is increasing gradually (31). However, more of these survivors develop a chronic lung dysfunction called BPD (31). Ever since BPD was described in 1967, various new prevention and treatment strategies have been implemented, such as antenatal and postnatal steroids, pulmonary surfactants, more rationally targeted oxygen saturation, and the use of noninvasive ventilation modes (3). However, among all the complications associated with preterm birth, BPD remains the first important and most frequent cause of neonatal mortality and morbidity (3, 31, 32). Preterm infants with BPD present with various short- and long-term adverse pulmonary and neurodevelopmental outcomes like asthma and repeated respiratory hospitalization, chronic cardiovascular disease in adulthood, and cerebral palsy or developmental delay (4, 6, 8). The main underlying pathogenesis for BPD is arrested alveolarization and vasculature resulting from the exposure of immature lungs to various perinatal factors that lead to a disrupted expression of genes critical for normal lung development (5–7).

To date, there is still a lack of effective intervention for BPD (3, 6, 7). Current therapy does not address the underlying lung structure and functional dysplasia (3, 10, 11). Recently, many studies have shown that resident and circulating stem cells play an important role in alveolar and lung vascular maturation, injury repair, and rationalizing inflammation (12, 15, 16). Human umbilical cord blood cells (UCBCs) are abundant in stem cells (32, 33). Compared with stem cells from other sources, UCB-derived stem cells present multiple advantages, including easier extraction, lower immunogenicity, and higher proliferation capacity without causing harm to the providers, and are believed to be the most potent cells for improving preterm lung development by regulating immune responses, alleviating inflammation, repairing injury, and reducing cell apoptosis by producing abundant paracrine factors (16–20, 32).

Our clinical studies and those of others have shown that cord blood-derived stem cell application is both safe and effective in the prevention of BPD in very preterm neonates (18–20, 27). Stem cells play a vital role in lung development, and their use for preventing or treating BPD has emerged as a promising therapy (15–21, 23, 27). However, the genetic heterogeneity between control and intervention groups may decrease the comparability between these groups, thereby reducing the statistical power of the therapeutic value of cord blood stem cells, especially in small sample trials (22). As various factors affect the complex molecular signals involved in the developing lung, the pathophysiological basis for BPD is manifold (6, 7). In this study, the intervention time point is the first 24 h after birth; our aim is to observe the preventive effect on BPD, and we will keep the postnatal medical treatment of twins to the maximum consistent according to the centers’ guideline. Furthermore, in the exclusion criteria, twins diagnosed with twin-to-twin transfusion syndrome confirmed by prenatal ultrasonography are also excluded. All these measures aim to minimize the environmental impact on the results. Genetic susceptibility to BPD is suggested by several studies on twins (22, 34), and there is an ongoing search for the role of genetic susceptibility to BPD (22, 35–37). Although in randomized control trials, baseline characteristics could be standardized or corrected for, it is difficult to evaluate or correct genetic predisposition. To treat very preterm monozygotic twins randomly could make the genetic predisposition distribution as similar as possible in two groups. As we know, monozygotic twins are regarded as the best research objects in clinical studies (38). Similar to a single-birth patient study, the different environment exposures or different growth rates will be balanced by the randomization and double-blinded characteristics between groups. As the genetic background in monozygotic twin premature infants is the same, the random enrollment of each of the twins in the placebo and MNC intervention groups will reduce the interference of genetic difference on assessing the protective effect of MNC on lung development and the outcome of BPD. This contributes to enhancing the statistical power of the therapeutic effect of cord blood stem cells and reducing the required sample size.

Previous preclinical studies showed that the effect of cord blood stem cell infusion in the prevention and treatment of BPD was determined mainly *via* anti-inflammatory and immune-modulatory functions. To investigate the underlying protective mechanisms of MNCs, we will perform single-cell RNA sequencing of peripheral white blood cells and test reactive oxygen species and inflammatory cytokines of serum. The results of single-cell RNA sequencing and inflammatory cytokines will facilitate an exploration of the complex molecular signals and alternation of immune cells involved in the repair of the developing lung, thereby revealing the mechanisms of how the cord blood MNC may modulate the immune cells and thus contributing to improving BPD.

This study is also designed to be double-blinded to minimize the bias resulting from treating physicians and researchers in collecting and analyzing data and following up patients.

In conclusion, since no studies have evaluated the effects of ACBMNC infusion on the prevention of BPD in very preterm monozygotic twins so far, this will be the first randomized, placebo-controlled, double-blinded trial to evaluate the efficacy of ACBMNC infusion as a prophylactic measure for BPD in monozygotic twin premature infants who have the same genetic background and investigate the underlying protective mechanisms *via* single-cell RNA sequencing and inflammatory cytokine tests. The results of this trial will provide valuable clinical evidence for translational application of cord blood cell therapy in very preterm infants.

## References

[1] Lancet Neonatal Survival Steering Team, Lawn JE Cousens S Zupan J. 4 million neonatal deaths: when? Where? Why? Lancet. (2005) 365(9462):891–900. 10.1016/S0140-6736(05)71048-5 15752534

[2] Stoll BJ Hansen NI Bell EF Walsh MC Carlo WA Shankaran S Trends in care practices, morbidity, and mortality of extremely preterm neonates, 1993–2012. JAMA. (2015) 314(10):1039–51. 10.1001/jama.2015.10244 26348753 PMC4787615

[3] Thébaud B Goss KN Laughon M Whitsett JA Abman SH Steinhorn RH Bronchopulmonary dysplasia. Nat Rev Dis Primers. (2019) 5:78. 10.1038/s41572-019-0127-7 31727986 PMC6986462

[4] Twilhaar ES Wade RM de Kieviet JF van Goudoever JB van Elburg RM Oosterlaan J. Cognitive outcomes of children born extremely or very preterm since the 1990s and associated risk factors: a meta-analysis and meta-regression. JAMA Pediatr. (2018) 172:361–7. 10.1001/jamapediatrics.2017.5323 29459939 PMC5875339

[5] Sahni M Bhandari V. Recent advances in understanding and management of bronchopulmonary dysplasia. F1000Res. (2020) 14:9. 10.12688/f1000research.25338.1 PMC7361502 32704351

[6] Hwang JS Rehan VK. Recent advances in bronchopulmonary dysplasia: pathophysiology, prevention, and treatment. Lung. (2018) 196:129–38. 10.1007/s00408-018-0084-z 29374791 PMC5856637

[7] Solaligue DES Rodríguez-Castillo JA Ahlbrecht K Morty RE. Recent advances in our understanding of the mechanisms of late lung development and bronchopulmonary dysplasia. Am J Physiol Lung Cell Mol Physiol. (2017) 313:L11010–L1153. 10.1152/ajplung.00343.2017 28971976

[8] Shepherd EG Nelin LD. Preterm birth, bronchopulmonary dysplasia, and long-term respiratory disease. Am J Respir Crit Care Med. (2017) 196:264–5. 10.1164/rccm.201703-0491ED 28762785

[9] Principi N Di Pietro GM Esposito S. Bronchopulmonary dysplasia: clinical aspects and preventive and therapeutic strategies. J Transl Med. (2018) 16:36. 10.1186/s12967-018-1417-7 29463286 PMC5819643

[10] Pierro M Thébaud B Soll R. Mesenchymal stem cells for the prevention and treatment of bronchopulmonary dysplasia in preterm infants. Cochrane Database Syst Rev. (2017) 11:CD011932. 10.1002/14651858.CD011932.pub2 29125893 PMC6485972

[11] Thébaud B. Stem cells for extreme prematurity. Am J Perinatol. (2019) 36:S68–73. 10.1055/s-0039-1691774 31238363

[12] Nitkin CR Rajasingh J Pisano C Besner GE Thébaud B Sampath V. Stem cell therapy for preventing neonatal diseases in the 21st century: current understanding and challenges. Pediatr Res. (2020) 87:265–76. 10.1038/s41390-019-0425-5 31086355 PMC6854309

[13] Ballen KK Gluckman E Broxmeyer HE. Umbilical cord blood transplantation: the first 25 years and beyond. Blood. (2013) 122:491–8. 10.1182/blood-2013-02-453175 23673863 PMC3952633

[14] Brunstein CG Wagner JE. Umbilical cord blood transplantation and banking. Annu Rev Med. (2006) 57:403–17. 10.1146/annurev.med.57.051804.123642 16409157

[15] Augustine S Avey MT Harrison B Locke T Ghannad M Moher D Mesenchymal stromal cell therapy in bronchopulmonary dysplasia: systematic review and meta-analysis of preclinical studies. Stem Cells Transl Med. (2017) 6(12):2079–93. 10.1002/sctm.17-0126 29045045 PMC5702524

[16] Walter J Ware LB Matthay MA. Mesenchymal stem cells: mechanisms of potential therapeutic benefit in ARDS and sepsis. Lancet Respir Med. (2014) 2:1016–26. 10.1016/S2213-2600(14)70217-6 25465643

[17] Behnke J Kremer S Shahzad T Chao CM Böttcher-Friebertshäuser E Morty RE MSC based therapies—new perspectives for the injured lung. J Clin Med. (2020) 9(3):682. 10.3390/jcm9030682 32138309 PMC7141210

[18] Ren Z Xu F Zhang X Zhang C Miao J Xia X Autologous cord blood cell infusion in preterm neonates safely reduces respiratory support duration and potentially preterm complications. Stem Cells Transl Med. (2020) 9(2):169–76. 10.1002/sctm.19-0106 31702120 PMC6988763

[19] Powell SB Silvestri JM. Safety of intratracheal administration of human umbilical cord blood derived mesenchymal stromal cells in extremely low birth weight preterm infants. J Pediatr. (2019) 210:209–13. 10.1016/j.jpeds.2019.02.029 30992220

[20] Ahn SY Chang YS Lee MH Sung SI Lee BS Kim KS Stem cells for bronchopulmonary dysplasia in preterm infants: a randomized controlled phase II trial. Stem Cells Transl Med. (2021) 10(8):1129–37. 10.1002/sctm.20-0330 33876883 PMC8284779

[21] Ren Z Xu F Zhang C Wang J Zhang Q Wen J Ten-year follow-up outcomes of a single intravenous infusion of autologous cord blood mononuclear cells in preterm neonates. Clin Transl Med. (2020) 10:e144. 10.1002/ctm2.144

[22] Lavoie PM Pham C Jang KL. Heritability of bronchopulmonary dysplasia, defined according to the consensus statement of the national institutes of health. Pediatrics. (2008) 122:479–85. 10.1542/peds.2007-2313 18762515 PMC4631604

[23] Ciccocioppo R Gibellini D Astori G Bernardi M Bozza A Chieregato K The immune modulatory effects of umbilical cord-derived mesenchymal stromal cells in severe COVID-19 pneumonia. Stem Cell Res Ther. (2021) 12(1):316. 10.1186/s13287-021-02376-9 34078447 PMC8170427

[24] Gary Cunningham F Leveno K Dashe J. Williams obstetrics. 26th edition, New York: McGraw Hill Medical (2022).

[25] Higgins RD Saade G Polin RA Grobman WA Buhimschi IA Watterberg K Evaluation and management of women and newborns with a maternal diagnosis of chorioamnionitis: summary of a workshop. Obstet Gynecol. (2016) 127(3):426–36. 10.1097/AOG.0000000000001246 26855098 PMC4764452

[26] Jensen EA Schmidt B. Epidemiology of bronchopulmonary dysplasia. Birth Defects Res Part A Clin Mol Teratol. (2014) 100(3):145–57. 10.1002/bdra.23235 PMC8604158 24639412

[27] Sun JM Song AW Case LE Mikati MA Gustafson KE Simmons R Effect of autologous cord blood infusion on motor function and brain connectivity in young children with cerebral palsy: a randomized, placebo-controlled trial. Stem Cells Transl Med. (2017) 6(12):2071–8. 10.1002/sctm.17-0102 29080265 PMC5702515

[28] National Health and Family Planning Commission of the People's Republic of China. Umbilical cord blood and stem cell bank management regulation (in Chinese). (2020). http://www.Moh.Gov.cn/zhuzhan/wsbmgz/201308/9da745d55fe749a2b82b95ee65294b55.shtml (Accessed June 1, 2020).

[29] Baker EK Wallace EM Davis PG Malhotra A Jacobs SE Hooper SB A protocol for cell therapy infusion in neonates. Stem Cells Transl Med. (2021) 10(5):773–80. 10.1002/sctm.20-0281 33405397 PMC8046110

[30] Gomella TL Cunningham MD Eyal F. Neonatology. 7th ed. New York: McGraw-Hill Education (2013).

[31] Walani SR. Global burden of preterm birth. Int J Gynaecol Obstet. (2020) 150:31–3. 10.1002/ijgo.13195 32524596

[32] Augustine S Cheng W Avey MT Chan ML Lingappa S Hutton B Are all stem cells equal? Systematic review, evidence map, and meta-analyses of preclinical stem cell-based therapies for bronchopulmonary dysplasia. Stem Cells Transl Med. (2020) 9(2):158–68. 10.1002/sctm.19-0193 31746123 PMC6988768

[33] Ren Z Xu F Wang J Zhong Z Wei W Wen J Safety and feasibility of umbilical cord blood collection from preterm neonates after delayed cord clamping for the use of improving preterm complications. Am J Transl Res. (2021) 13(5):4553–60. 34150035 PMC8205698

[34] Wang H St Julien KR Stevenson DK Hoffmann TJ Witte JS Lazzeroni LC A genome-wide association study (GWAS) for bronchopulmonary dysplasia. Pediatrics. (2013) 132(2):290–7. 10.1542/peds.2013-0533 23897914 PMC3727675

[35] Yu KH Li J Snyder M Shaw GM O'Brodovich HM. The genetic predisposition to bronchopulmonary dysplasia. Curr Opin Pediatr. (2016) 28(3):318–23. 10.1097/MOP.0000000000000344 26963946 PMC4853271

[36] Bhandari V Bizzarro MJ Shetty A Zhong X Page GP Zhang H Familial and genetic susceptibility to major neonatal morbidities in preterm twins. Pediatrics. (2006) 117(6):1901–6. 10.1542/peds.2005-1414 16740829

[37] Bhandari V Gruen JR Jang KL Göpel W Hallman M Lavoie PM. Genetics of bronchopulmonary dysplasia: when things do not match up, it is only the beginning. J Pediatr. (2019) 208:298–9. 10.1016/j.jpeds.2019.01.014 30737034

[38] Polderman TJ Benyamin B de Leeuw CA Sullivan PF van Bochoven A Visscher PM Meta-analysis of the heritability of human traits based on fifty years of twin studies. Nat Genet. (2015) 47(7):702–9. 10.1038/ng.3285 25985137

