# Supplementary material for: Autologous cord blood mononuclear cell infusion for the prevention of bronchopulmonary dysplasia in very preterm monozygotic twins: A study protocol for a randomized, placebo-controlled, double-blinded multicenter trial
Source: Front Pediatr. 2022 Dec 9;10:884366. doi: 10.3389/fped.2022.884366 (PMC9780444; doi:10.3389/fped.2022.884366)
Supplement: Supplementary file 2 [file Table2.docx]

Additional file 2: Contents and points of data capture: Standard Protocol Items: Recommendations for Interventional Trials (SPIRIT) schedule of enrolment, interventions, and assessments

| Visit | Screening | Intervention | Follow up | | |
| --- | --- | --- | --- | --- | --- |
|  | V1 | V2 | V3 | V4 | V5 |
| Time point | born | Within 24 hours after birth | 36 weeks of postmenstrual age | corrected age of one years old | corrected age of two years old |
| Informed consent form | √ |  |  |  |  |
| Screening the subject | √ |  |  |  |  |
| Demographic information | √ |  |  |  |  |
| Inclusion/exclusion criteria | √ |  |  |  |  |
| Get random number | √ |  |  |  |  |
| Cord blood process |  | √ |  |  |  |
| Vital signs | √ | √ | √ |  |  |
| Pulmonary surfactant replacement |  | √ |  |  |  |
| Mechanical ventilation | √ | √ |  |  |  |
| Oxygen therapy | √ | √ | √ |  |  |
| Arterial blood oxygen saturation | √ | √ |  |  |  |
| Blood gas | √ | √ |  |  |  |
| Safety outcomes |  | √ | √ | √ | √ |
| Record adverse events |  | √ | √ | √ | √ |
| Surgical closure of patent ductus arteriosus |  | √ | √ |  |  |
| Blood products use |  | √ | √ |  |  |
| Bronchopulmonary dysplasia |  |  | √ |  |  |
| other preterm complications |  |  | √ |  |  |
| Duration of hospitalization |  |  | √ |  |  |
| Anthropometric Characteristics |  |  | √ | √ | √ |
| Respiratory Outcomes |  |  |  | √ | √ |
| Neurodevelopmental outcomes |  |  |  | √ | √ |
